# Business as (un)usual: A qualitative study of clerkship experiences during a health crisis

**DOI:** 10.1111/medu.14787

**Published:** 2022-03-07

**Authors:** Laerke Marijke Noerholk, Karlen S. Bader‐Larsen, Anne Mette Morcke, Anishan Vamadevan, Lisbeth Anita Andreasen, Jesper Hastrup Svendsen, Hanne Jørsboe, Martin G. Tolsgaard

**Affiliations:** ^1^ Copenhagen Academy for Medical Education and Simulation (CAMES) Copenhagen University Hospital – Rigshospitalet Copenhagen Denmark; ^2^ Centre for Educational Development Aarhus University Aarhus Denmark; ^3^ Department of Clinical Medicine, Faculty of Health and Medical Sciences University of Copenhagen Copenhagen Denmark; ^4^ Department of Cardiology Copenhagen University Hospital ‐ Rigshospitalet Copenhagen Denmark; ^5^ Nykøbing Falster Sygehus, Region Zealand Nykøbing Falster Denmark; ^6^ Centre for Fetal Medicine, Juliane Marie Centre Copenhagen University Hospital ‐ Rigshospitalet Copenhagen Denmark

## Abstract

**Introduction:**

During a health crisis, hospitals must prioritise activities and resources, which can compromise clerkship‐based learning. We explored how health crises affect clinical clerkships using the COVID‐19 pandemic as an example.

**Methods:**

In a constructivist qualitative study, we conducted 22 semi‐structured interviews with key stakeholders (i.e. medical students and doctors) from two teaching hospitals and 10 different departments. We used thematic analysis to investigate our data and used stakeholder theory as a sensitising concept.

**Results:**

We identified three themes: (1) emotional triggers and reactions; (2) negotiation of legitimacy; and (3) building resilience. Our results suggest that the health crisis accentuated already existing problems in clerkships, such as students' feelings of low legitimacy, constant negotiation of roles, inconsistencies navigating rules and regulations and low levels of active participation. Medical students and doctors adapted to the new organisational demands by developing increased resilience. Students responded by reaching out for guidance and acceptance to remain relevant in the clinical clerkships. Doctors developed a behaviour of closing in and focused on managing themselves and their patients. This created tension between these two stakeholder groups.

**Conclusion:**

A health crisis can critically disrupt the hierarchical structure within the clinical clerkships and exacerbate existing conflicts between stakeholder groups. When medical students are not perceived as legitimate stakeholders in clinical clerkships during a health crisis, their attendance is perceived as unnecessary or even a nuisance. Despite increased student proactiveness and resilience, their roles inevitably shift from being doctors‐to‐be to students‐to‐be‐managed.

## Abstract 2

This study reports that when medical students are not perceived as legitimate stakeholders in clinical clerkships during a health crisis, their roles shift from being doctors‐to‐be to students‐to‐be‐managed.

## 1 INTRODUCTION

During a health crisis, clinical clerkships are completed under exceptional conditions, where all formal and informal educational practices are affected. For a hospital department to remain functional during a health crisis, the various needs of different stakeholder groups necessitate the renegotiation of priorities between service and education.^1^, ^2^, ^3^ A health crisis is defined by the Council of the European Union as ‘any crisis or serious incident arising from a threat of human, animal, plant, food or environmental origin, having a health dimension and which requires urgent action by authorities’.^4^ Epidemics and—more rarely—pandemics represent one type of health crisis that develops when the infectious agent is new and unknown and the health care systems are unprepared or poorly equipped for controlling it.

Social distancing is a key element in the containment of infectious diseases. It played a major role in past outbreaks such as the 1918 influenza pandemic.^5^ However, upholding social distancing and the associated use and distribution of personal protective equipment is difficult in a hospital setting, and it represents a unique challenge in clinical clerkships. Medical students are under supervision from senior colleagues and rarely manage patient care independently. In most cases, this means that social distancing is compromised by the addition of a medical student, who by some may be considered dispensable for clinical service. The prioritisation of resources during a health crisis puts medical students in a vulnerable position, where they may either be included as an additional resource (for example, as was seen during the Copenhagen 1952 poliomyelitis epidemic, where medical students provided manual ventilation^6^) or they may be left out of the clinical context with their education at risk of being overlooked.^7^ Learning through clinical clerkships and interaction with real patients is a core element of medical students' education.^8^, ^9^ Clerkship learning is often viewed as a social process; that is, interaction and collaboration with and observation of stakeholders in hospital departments are key elements for student learning to occur.^10^ Yet, challenges regarding this learning format are well known, for example, that the effectiveness of teaching and learning has been characterised as random and opportunistic^11^, ^12^ and that students often are unsupervised and receive inadequate feedback.^13^ However, little is known about the impact of a health crisis, such as the COVID‐19 pandemic with its high level of social distancing, on the quality of clinical clerkships. Although the balance between service and training in clerkships is also described as challenging outside times of crisis,^14^ health crises provide unique threats to and insights into clinical education as everyday organisational systems are broken down in order to handle an urgent and extraordinary situation.^15^ Whether health crises are triggered by pandemics,^7^, ^16^, ^17^ poverty,^18^ natural disasters^19^ or even economic crises,^20^ the prioritisation of resources is important to address, as it may be difficult to navigate for students and educators alike.

This study was inspired by stakeholder theory. Originally a theory of organisational management and business ethics,^21^ its use in health care is nevertheless progressing.^22^, ^23^, ^24^, ^25^ Recent research has illustrated how conceptualising hospital departments as business entities allows for the development of a nuanced view of why health care stakeholders prioritise as they do in times of disruption.^26^ Additionally, the illumination of the complicated dynamics between stakeholders provide a better understanding of concepts such as competence or power in a clinical context.^27^ We aimed to investigate what insights the use of stakeholder theory would provide when used to analyse behaviours and patterns developed by different stakeholder groups in clinical clerkships during a health crisis.

In 1984, Freeman introduced stakeholder theory, defining stakeholders as ‘any group or individual who can affect or is affected by the achievement of the organization's objectives’.^28^ The theory implies looking at all stakeholders and their perception of value in addition to the values important to the organisation in question. This leads to the creation of economic value as well as ensuring the well‐being of the involved parties.^21^, ^29^ When viewing clinical clerkships from a stakeholder theory point of view, medical students can become a potential area of conflict as they do not produce immediate value for the organisation (in this case, for patient treatment or the department itself).^29^ Particularly, a conflict may arise, if other stakeholders perceive students as lacking justification in the clinical setting, and students feel they have a right to participate for educational reasons. Thus, the identification and understanding of stakeholders' perceptions of what is valuable and what is counterproductive can be important in the analysis of health crisis management and in the education of medical students in a system where many other priorities are at play.^15^, ^30^, ^31^ In addition, stakeholder theory may help identify the power, legitimacy and urgency of different stakeholders including their underlying interests and alliances.^32^, ^33^, ^34^ Not all stakeholders are entitled to the same amount of influence on the decisions made, rather the more influential have more power of decision making. Consequently, some decisions regarding a group of stakeholders may not be perceived as being reasonable by all,^32^ and it is therefore important to identify stakeholders' various views to counteract potential conflicts in the clinical workplace.^35^

To develop a better understanding of stakeholders' experiences with clinical clerkships during health crises, we used the current COVID‐19 pandemic as an example of a health care system in crisis. As such, we set out to answer the following research question: How does a health crisis impact medical students' clinical clerkships? By acknowledging that health crises are recurrent events, it is important to better understand the dynamics between medical students and clinicians to inform the management of clinical clerkships in future health crises.^31^

## 2 METHODS

### 2.1 Design

In this exploratory study, we worked within a constructivist qualitative paradigm^36^ and used thematic analysis to investigate the perceptions of medical students' clinical clerkship training during a health crisis from the perspectives of different stakeholders.^37^, ^38^ We selected doctors and medical students as the representative stakeholders to be included in this study, as we believed they are most involved in the clerkships of medical students. Initially, LMN and KBL coded the entire dataset and subsequently used stakeholder theory as a sensitising concept to enhance and prime our understanding of the underlying themes in our data.^39^

### 2.2 Context

Participants were recruited from two Danish teaching hospitals and 10 different clinical departments. These hospitals represented a smaller rural hospital and a large highly specialised tertiary hospital, which allowed for exploration of different clerkship activities based on hospital size and patient uptake. The rural hospital received COVID‐19 patients at the beginning of the pandemic but did not have a unit for specialist treatment at the time of data collection; that is, if patients were diagnosed with COVID‐19, they were transferred to another hospital in the region. The large hospital received and treated COVID‐19 patients in specialised treatment units throughout the pandemic.

### 2.3 Participants

Participants were recruited using a purposive sampling strategy,^40^ where two members of the research group (HJ and MGT) initially appointed key stakeholders from the following groups: medical students and supervising clinicians. In the group of clinicians, the participants were identified based on HJ's and MGT's knowledge of their clinical areas and their attitudes towards the education of medical students during the COVID‐19 pandemic to allow for a broad variation of opinions. For example, some doctors were identified due to their proactiveness to keep medical students in the clinical setting and some were identified due to the opposite. After the initial appointment process, we used a snowball sampling technique, where already appointed participants identified new participants that were contacted by LMN and invited to participate.^40^ Eleven clinicians, including four professors, four senior doctors and three junior doctors, were interviewed for the study, and their specialties covered gynaecology/obstetrics (three), thoracic surgery (one), anaesthesiology (one), cardiology (two), internal medicine (two) and orthopaedic surgery (two). All of the professors were affiliated with the University of Copenhagen.

The medical students were chosen based on a convenience sample; that is, the supervising clinicians shared the email addresses of medical students in their clerkship stays with LMN, who invited them to participate.^40^ The medical students were in their fourth to sixth year of training at the University of Copenhagen (where the total duration of the medical studies is 6 years), and participation was optional. The clerkships covered a broad range of clinical specialities (i.e. obstetrics/gynaecology, internal medicine, cardiology, orthopaedic surgery, neurosurgery, general anaesthesiology, paediatrics and urology) and students spent from 2 to 5 weeks in each department. Eleven medical students were interviewed for the study.

### 2.4 Data collection

Initially, LMN developed a semi‐structured interview protocol to help explore how the training of medical students in clinical clerkships was being undertaken, triaged and prioritised (see Appendix S1).^41^ Subsequently, it was reviewed by MGT and KBL and, with their feedback, was adjusted to fit both stakeholder groups. Additionally, an experienced qualitative researcher reviewed the interview protocol and provided feedback. However, the reviewer recommended no additional changes.

The primary investigator, LMN, conducted individual interviews during late April and May 2020, that is, when the first wave of the COVID‐19 pandemic was beginning to decrease. This allowed for our participants to look back and reflect on their experiences from the peak of the first wave as well as their current experiences. As the interviews had to be conducted quickly to capture fresh perspectives, the team aimed to conduct rich interviews with as many participants as possible in a short period.

Coding was conducted in three cycles until no new codes were seen in the data. As Varpio et al. argue that true theoretical saturation may not be entirely possible to reach,^42^ the research team felt that the quality of the interviews, aim of the study and multiple coding cycles allowed the data collection to justifiably end. Due to social distancing restrictions, some interviews were undertaken using online media (i.e. Zoom, Skype and Facetime), and some interviews were undertaken face‐to‐face in settings where proper distance could be maintained.

### 2.5 Data analysis

All interviews were transcribed verbatim and analysed by LMN and KBL. We conducted a thematic analysis following the model by Braun and Clarke^37^ by (1) familiarising ourselves with the data, (2) generating initial codes, (3) searching for themes, (4) reviewing themes, (5) defining and naming themes and (6) reporting. After completing Step (1), in Step (2), the 22 interviews were coded in three phases. After LMN and KBL coded the first seven interviews, they developed an initial coding scheme. This coding scheme was used to code the next eight interviews, with the addition of new codes. LMN and KBL thus adjusted and developed the coding scheme further. At this stage, the tentative coding scheme was checked and commented on by AV, who used it to code four interviews (two doctors and two medical students). AV provided comments to each code and discussed them with LMN and KBL, which fine‐tuned the coding scheme further. The revised scheme was used for the coding of the last seven interviews by LMN and KBL.

After both KBL and LMN coded all 22 interviews, they developed the final coding scheme. During this process, they generated 13 codes that LMN used to re‐code the entire dataset. She managed the final coding and extraction of codes using a web‐based qualitative analysis program (www.dedoose.com). Two senior medical education scientists (MGT and AMM) received the final coding scheme and two interviews (one doctor and one student) with LMN's coding to check if the codes resonated with them. Based on their feedback, LMN, KBL and AV discussed the findings and how the codes could be compiled into themes. Especially, the concepts of the stakeholder's power (to influence the organisation), legitimacy (of the stakeholder's relationship with the organisation) and urgency (of the stakeholder's claim on the organisation) were used to guide the analysis.^34^ Finally, they constructed three themes that were apparent and consistent throughout the dataset.

### 2.6 Reflexivity

The research group is composed of junior and senior researchers with different backgrounds and professions. LMN is a junior doctor with 4 years of clinical experience from various departments. She is now a PhD fellow, and her studies have focused on clinical clerkship learning and collaborative learning. As a medical doctor with educational responsibilities, she has insights into the perspectives of both stakeholder groups included in this study. KBL is an American medical education researcher with a background in Nutritional Sciences and ample qualitative experience. Her perspectives have especially strengthened the methodological rigour of the study. AV is a medical student at the same university as the medical students in our study and is doing educational research at our institution. He contributed as a student representative with unique insights into the dataset as he had recent experience with participation in clinical clerkships both before and during the data collection period. MGT is an obstetrician and medical education scientist, LAA is an obstetric resident and has a PhD in medical education, AMM is the director of a large educational unit in Denmark, HJ is a consultant doctor working with organisational structure and JHS is a professor in cardiology and Head of Department of Clinical Medicine at the University of Copenhagen, which is responsible for all clinical clerkship programmes conducted in this site. These various backgrounds and experiences in the author group allowed us to interpret the data from different angles and with distinct insights.

### 2.7 Ethical approval

This study was reported to the Regional Ethics Committee of the Capital Region of Denmark and was deemed exempt from review, Protocol no: H‐20026744. All participants were briefed on the details of the study, verbally agreed to be interviewed and signed informed consent forms.

## 3 RESULTS

Through our thematic analysis, we identified three main themes: (1) emotional triggers and reactions; (2) negotiation of legitimacy; and (3) building resilience.

### 3.1 Emotional triggers and reactions

When students experienced the organisational context of a health crisis, they were emotionally triggered by the rejection they experienced from doctors. They described a feeling of neglect and a feeling of failure because they were not allowed full participation and optimal learning conditions. This consequently reduced their perceived power to influence the organisation, and they reacted to their feeling of being left behind by being more proactive:

When they [the doctors] (…) had to go to their outpatient clinic or something, you had to be very proactive and sometimes it felt like you had to demand to join them (Student 7).

However, students felt challenged on how to behave proactively when social distancing guidelines had to be followed:

The doctors were under more pressure and they had less time to explain and teach. Those things mean that you have to be really, really proactive and being proactive is really something with body language. It means to physically lean in and show that you're interested. But you had to keep a distance and move away (…) it was like really difficult for me to find out how to do it (Student 6).

One challenge was that the students felt a reduced educational value of their clerkships, and they were worried that a lack of skills and knowledge would impact their future practice:

… I think it's been a great disappointment for a lot of us students because we've felt that it has had an influence on our learning and on our clerkship which we won't get another shot at for a while (Student 1).

Despite the fear of losing necessary competencies, students seemed to accept that the situation was extraordinary and that their learning was perceived as being less urgent than patient management:

There's no focus on our learning right now; I mean, it's a bit secondary at the moment, right? (Student 1).

During the first wave of the pandemic, the number of participants permitted to join physical meetings and conferences was reduced in both hospitals. Because the students were instructed by the university to be present at the departments but were not allowed to take part in daily meetings, it affected their sense of intrinsic value and made them feel illegitimate:

I think it's the feeling of just standing there and not being allowed to join (the meeting), I really hate that … I mean, the feeling that I'm making a fool of myself really ‐ just standing there, in the back of the room and not taking part (Student 3).

They described strong feelings of guilt and needed to confirm with one another that they were not to blame for being present:

So that you could put into words that you weren't the problem – others have experienced the same thing … It's probably just the attitude that exists towards us (Student 6).

Medical students were also triggered by the unpredictability they faced when entering the clinical setting, which was perceived as frustrating and very hard to navigate:

You would say: ‘I would like to see this or attend rounds or do a rectal examination’ and then you would get different answers depending on who you asked … we had no clear indication of what was demanded of us and what we could demand of them when it came to getting our education (Student 7).

This inconsistency impacted students' general motivation to participate in the clinical setting negatively, and the general confusion about what their role and responsibilities should be during a health crisis made them feel unwelcome:

Well, the first day when we attended the morning meeting some of them [doctors] asked us directly: ‘Are you supposed to be here?’ and ‘We didn't think you were allowed to be here.’ And then it was just a feeling that no one asked us to join them because, well, there wasn't really the time nor the room for it, unfortunately (Student 2).

For some students, this translated into a feeling of inadequacy, which affected the way they understood themselves in the clinical setting:

Well, I don't even go [to the morning meeting] anymore, I'm afraid of the defeat (Student 3).

On the other hand, some doctors were triggered by the medical students' presence when they themselves had to deal with new tasks and responsibilities as well as cancellations of their own planned educational activities. This caused them to react more dismissively towards students:

And then the ambivalence that they [the medical students] have to come in and learn when all of our [the doctors'] teaching sessions have been cancelled, all of our education, all the fun parts of being a doctor have been cut, but the medical students have had them anyway (Clinician 1).

This conflict affected the students, and they felt that they had to establish a good reputation and behave appropriately:

So, it was a little hard to navigate the whole: ‘How do I keep being proactive but still respect the rules?’ Because you want to prove to them [the doctors] that you can do it (Student 6).

Doctors were aware that the conditions under which the students trained were far from optimal and recognised that students were lost when normal gatherings, like department meetings, were cancelled. This was believed to influence both the students' motivation and their learning in general, as participation in both formal and social activities was seen as crucial for students' professional development:

It's not so much the lack of interaction [with patients, doctors etc.], it's more [the lack of] the informal, the less structured things and the fun (Clinician 4).

### 3.2 Negotiation of legitimacy

Medical students displayed conflicting perspectives on whether they had a right to take part in patient consultations and daily activities in the department. Some felt that they had a right to learn despite the circumstances, whereas others felt that social distancing measures were more important than ensuring adequate teaching conditions. To feel legitimate during the health crisis, the students felt they needed to justify their presence:

Because society has been so focused on extra sources of infection and that the [patients'] relatives haven't been allowed in the hospital, that's made it morally hard for me [to accept] that I was there. For me, I've felt that I had to justify my presence (Student 8).

This need to actively defend one's presence also translated into students questioning whether their attendance could be perceived as transgressive behaviour:

There has been an uncertainty connected with us moving around [the department] sometimes; like: ‘Am I getting too close? Is this crossing the line?’ (Student 5).

With this question lingering at the back of their minds, students increasingly felt they needed to contribute to preserve their legitimacy. However, it was difficult to accomplish, and senior staff expressed that it was a general problem, even before the crisis:

We haven't really succeeded in integrating their [the students'] functions in the daily management. We find that the departments that have the highest scores on student evaluations are the ones where the students are expected to actively engage and contribute (Clinician 3).

The cooperation between the university and the clinical departments seemed to be lacking common ground in creating an understanding of why the medical students were present and to what extent they were expected to participate during a health crisis. As university stakeholders have the highest official power over medical students (i.e. the approvement of a satisfactory course), the medical students had to follow the university's directions and attend the department's activities in order to continue their medical training. However, this managerial decision was difficult to put into practice, because the stakeholders of the department had the final say in whether to allow students to participate or not. This created a conflict between the university and the departments, leaving the students with the feeling that their presence was illegitimate:

I remember a morning meeting and one of the senior doctors said: ‘Are the students back again?’ and you didn't really know if you were welcome. They [the doctors] didn't even know what happened with the university and if we were supposed to be there or not (Student 11).

Doctors echoed these problems:

If we are to have them [students attending clinical clerkships] then we need to do it properly. It is rather unsatisfactory having students present and then not being able to provide them with adequate education (Clinician 1).

These experiences made the students increasingly aware of the medical hierarchy, and some felt that they took up a spot that was not theirs to take as well as a reduced power to influence their own participation in the clinical setting.

None of the students described problems with patient collaboration. They did, however, describe that patient concerns were sometimes mentioned as a reason for rejecting them (e.g. when relatives were not allowed to participate in consultations, neither should students). Some doctors also mentioned that they struggled with allowing students a place over relatives:

It's obvious that when you try to limit the number of people entering, then it creates an unbalance if you say: ‘We prioritize that the medical students should join in, but you are not allowed to visit your own family member’ (Clinician 5).

As such, the traditional medical hierarchy seemed to be magnified during the crisis. The value of medical students decreased, and doctors prioritised being able to attend to their own and their patients' interests, which they considered the most urgent:

I'd be angry about being excluded too since it's training they're [the students are] entitled to. But at the moment you aim for the core tasks … it's the patient, then it's the relatives and then it's the students (…) (Clinician 2).

### 3.3 Building resilience

Working during a health crisis impacted both stakeholder groups tremendously. Our analysis showed that building up resilience, understood as stakeholders' ways to adapt to the new challenges as well as uphold the organisation's core tasks, was essential for both doctors and students to handle the new organisational environment. However, the two stakeholder groups managed it differently. Students did their best to continuously interpret and adjust to the new rules to remain relevant and legitimate in the departments. After the initial experiences of feeling rejected, some students coped by doing everything in their power to preserve value in the department:

Let's try to start fresh and really go all‐in on adhering to those rules, so we'll be in as good a standing as possible (Student 6).

They developed their ability to reach out for guidance and perceived the task of acquiring other stakeholders' acceptance as their personal responsibility. As a result, their own value perception was encapsulated in a clear desire to be allowed to participate in the department's activities and take on a role that reflected their current competency level. By doing so, they saw the opportunity to adapt to the crisis by reducing the burden for doctors, staying involved and gaining crucial experience at the same time:

I find it extremely important that we, as students, take part. Also, it's not certain that this will happen only once during our lifetime. So, if we, starting from medical school, can gain experience saying: ‘How did we react and feel during this and what can be done better the next time a pandemic strikes? Or if there's a major rearrangement of the health care system …?’ (Student 7).

Doctors recognised these efforts, and some expressed that continuing medical students' education should be considered important for the survival and continuation of the entire organisation:

You shoot yourself in the foot if you don't keep educating and can't keep up [with clinical tasks]. So, it's simply something we have to do. And it's proved to be feasible in a fair manner (Clinician 8).

However, in several clinical settings, we found that doctors built up their resilience by developing a behaviour of closing in, where they mainly focused on managing themselves and their patients. In retrospect, they described students as simply being ‘forgotten’ in crisis management, and consequently, students were left out of the equation. They highlighted the importance of knowing and recognising the students because when students were not physically visible in the departments, their urgency diminished as they were unrecognisable and unrelatable:

Since we haven't had our morning meetings, it [the relation to the students] has vanished as there haven't been any faces that you could relate to and then sometimes it's harder to say: ‘Hey you, won't you join me?’ when you don't know who the person is (Clinician 2).

For some doctors, the combination of new schedules daily and the increased proactive behaviour of students could become a nuisance:

I had planned a lot [of educational activities] and then, when it came down to it, it wasn't feasible and then they [the students] called me all the time … and said: ‘Where should I go then?’ and ‘I'm not allowed to be here’ or ‘There are no patient files to record’ (…) They [the students] needed a lot of ‘babysitting’ (Clinician 1).

This emphasises the perception of the value and urgency of student participation during a health crisis because their attendance could be strenuous to manage for doctors. Whether the organisations' response to a crisis should be to regard students as critical resources or not was debated among doctors; however, some also expressed wishes for students to return as they were seen as motivators for constantly improving the clinical environment:

Well, actually, they [the students] do it [contribute] all the time because there are things we suddenly have to follow up on, right, just to make sure that we are up‐to‐date on things (Clinician 6).

This ambiguity of viewing students as non‐essential stakeholders on one hand and as motivators for the clinical educational environment on the other hand explains why students felt they needed to develop an increasingly proactive behaviour with a tougher attitude to succeed in their clerkships during a health crisis. Yet, doctors may not have been able to fully appreciate these efforts.

## 4 DISCUSSION

This study highlights key aspects of how clinical clerkship training of medical students is (not) prioritised during a health crisis. Emphasising medical students' active participation as a central value in clinical clerkship,^8^, ^43^ our analysis identified both organisational and individual challenges in the management of medical students and their continued education.

First, our data indicate a value system in the organisational context of a hospital, where students' learning seems to be underprioritized significantly during a health crisis. This is to be expected,^2^, ^16^ but it is notable that students easily accept their training as being of less importance than patient care, research projects, management of regular staff and so on. This may be because students as stakeholders possess no ‘freedom of choice’ and are relatively powerless, as they cannot seek clerkship elsewhere. They are bound by the formal commitments and agreements between hospital departments and the university.^32^, ^44^ From a stakeholder perspective, students are ‘caught in the middle’ to some extent, because they use resources like a customer (i.e. like a primary stakeholder^44^) but do not contribute with any immediate local value. Instead, they merely offer value on a societal scale by training as part of the future workforce. Clinicians, who can be considered local primary stakeholders, were obviously more interested in how to ensure high‐quality clinical service. Moreover, in this context, universities and governmental institutions are secondary stakeholders^44^ that have a high influence on departments during crises without having a direct economic stake. A department's adherence to regulations addressed by these institutions is a crucial part of their crisis management strategy and may overrule other objectives, such as medical education.^30^ In other words, what normally is a mutually beneficial environment where students draw on resources for training while providing value in service had now shifted balance as a consequence of the health crisis.

Second, problems related to student transition from pre‐clinical to clinical training as well as transitions within clinical training are well known^8^, ^45^, ^46^ but seem to be exacerbated and disrupted during a crisis. Organisational disruptions can be triggered by external agents, such as health or economic crises or natural disasters,^3^, ^7^, ^18^, ^19^, ^20^, ^47^ or internal agents, such as changes in educational structure and instructional design.^26^ When examining how an external agent (a pandemic) affected clinical clerkships, we found patient concern was part of the clinicians' arguments against student participation. We have previously documented the same pattern of reactions but provoked by internal agents that disrupted a department's educational infrastructure (e.g. the introduction of dyad learning in a clinical clerkship).^26^ The resulting conflicts between stakeholder groups are believed to induce stress and anxiety in students, which has the potential to impact learning negatively.^46^

Third, we found that medical students are deprived of the opportunity of developing a meaningful role during clerkship training when they are not included as accepted members of the team. Students' experiences during clerkships are based on a socialisation process, which should lead to a better understanding of their current roles as students as well as their future roles as doctors.^48^ This is believed to be crucial for medical students' professional identity formation.^49^ Our data point to this socialisation process being deeply impaired during a health crisis. Additionally, students in ‘normal’ clerkships report spending an unexpected amount of time navigating the system,^48^ and our findings indicate that these efforts went from difficult but feasible to insurmountable. Recent research emphasises mistreatment as a potent shame trigger, which can make medical students feel unworthy of being present in clerkships.^50^, ^51^ This could be another reason for students' acceptance of their training being underprioritized. Because medical students' professional identity formation process has a main goal of making them ‘think, act and feel like a physician’,^52^ our results suggest that they may already identify with doctors to a degree that make them question their own legitimacy as students.

After the resolution of a health crisis, a new norm will surface, but the question is when and how this new norm is going to form. More importantly, the findings made in our study are likely to be reproduced in future health crises as medical education strategies have a tendency to repeat themselves.^53^ The conflicts demonstrated between stakeholder groups and the negative effects reported in our study as well as in numerous others^1^, ^7^, ^54^, ^55^, ^56^, ^57^, ^58^, ^59^ may at first be perceived as the result of a dysfunctional or collapsed health care system. However, the mechanisms may also demonstrate the resilience of our current educational systems that it responds adequately to a health crisis that requires prioritisation between imminent needs (patient care) and postponable needs (education) for a period of time before returning to a new normal.^60^ Finding that suitable balance between flexibility and stability in an organisation in crisis is crucial.^35^ Our findings point to the necessity of a clear (down‐)prioritisation of less urgent tasks for medical students before a crisis occurs in order to diminish general stakeholder confusion and mistreatment during a crisis. Such a crisis plan should be formulated in advance by universities and hospitals to identify and plan for which educational activities should be sustained, dropped or revised early in crisis management.^61^ For example, the organisation may operate most efficiently without its less powerful stakeholders—the medical students—for a limited period of time. However, a post‐crisis plan that ensures how medical students' training needs are met in the long term is also warranted, as their claim for training is both legit and will eventually become urgent should a crisis be prolonged. Stakeholder identification and subsequent analysis^32^, ^34^ provides an approach for future research and development of these plans, as it helps clerkship managers identify how, why and when to attend to different groups of stakeholders. This could be an important step towards better and more adaptable crisis management in medical education in the future, which has been called upon from the medical education society.^62^

### 4.1 Strengths/limitations

By recruiting participants from (1) different clinical specialties, (2) various clerkship years and (3) one small rural versus one large central hospital, this maximum variation approach allowed us to search for commonalities in our dataset across a heterogeneous population, thereby increasing the transferability of our results. In the data analysis, we used stakeholder theory as a sensitising concept and followed a protocol with two coders coding the entire dataset and ongoing development of a coding scheme through discussion and adjustments. Additionally, the author group consisted of a broad range of clinicians, education scientists, a medical student and administrative faculty. This supports the trustworthiness of our data analysis. We chose to interview medical students and doctors only, as they were believed to have the most influence on the clerkships in our context; however, our data lack the perspectives of nurses, other health care providers and patients, who all play an important part in medical students' education in the clinical setting. Moreover, medical students may participate differently in the hospitals' communities of practice in other countries, and data collected during a health crisis may not be transferable on a one‐to‐one basis to other types of crises, such as natural disasters and terrorist attacks.^3^

## 5 CONCLUSION

A health crisis can critically disrupt the hierarchical structure within the clinical clerkships and exacerbate existing conflicts between stakeholder groups. When medical students are not perceived as legitimate stakeholders in clinical clerkships during a health crisis, their attendance is perceived as unnecessary or even a nuisance. Despite increased student proactiveness and resilience, their roles inevitably shift from being doctors‐to‐be to students‐to‐be‐managed.

## CONFLICT OF INTEREST

The authors declare that they have no conflicts of interest.

## AUTHOR CONTRIBUTIONS

LMN was responsible for writing the study protocol, the data collection, data analysis and translation of quotes and is the first author of the paper. KBL was responsible for data analysis and translation of quotes and is a co‐writer of the paper. AMM was responsible for co‐writing the study protocol and data analysis and is a co‐writer of the paper. AV was responsible for data analysis and is a co‐writer of the paper. LAA was responsible for translation of quotes and was a co‐writer of the paper. JHS and HJ were co‐writers of the study protocol and the paper. MGT was responsible for planning the study, co‐writing the study protocol and data analysis and is a co‐writer of the paper.

## ETHICAL APPROVAL

This study was reported to the Regional Ethics Committee of the Capital Region of Denmark and was deemed exempt from review, Protocol no: H‐20026744. All participants were briefed on the details of the study, verbally agreed to be interviewed and signed informed consent forms.

## Supporting information




**Data S1.** Supporting Information
