# Supplementary material for: Business as (un)usual: A qualitative study of clerkship experiences during a health crisis
Source: Med Educ. 2022 Mar 7;56(8):805–14. doi: 10.1111/medu.14787 (PMC9543617; doi:10.1111/medu.14787)
Supplement: Supplementary file 1 — Data S1. Supporting Information [file MEDU-56-805-s001.docx]

**Interview protocol for medical students:**

**Open question no. 1: *(General)***

1. What is it like to participate in a clinical clerkship during the COVID-19 pandemic?

1. Has anything changed? If so, how is it different compared to earlier experiences?
2. How are you welcomed by your peers?
3. How are you welcomed by the doctors of the department?
4. How are you welcomed by the patients?

**Open question no. 2: *(Classification of clinical activities)***

2. Could you describe your last day(s) in your clinical clerkship?

1. Which activities do you participate in during your clerkship? What do you do on your own and what do you do in collaboration with others?
2. Are there any precautionary measures that you have to adhere to?
3. Do you feel that the recommendation to ”keep your distance” affects your activities in the clinical setting?
4. Have you experienced being rejected in tasks or assignments which you believe you normally would have been able to participate in or undertake?
5. Have you experienced being offered any tasks or assignments which you believe you normally would not have been able to participate in or undertake?
6. How do you normally structure your study activities during a clinical clerkship? What do you do at the hospital and what do you do at home or in other places? Has anything changed during the COVID-19 pandemic?

**Open question no. 3: *(Interactions with peers)***

3. How do you interact with your peers at the moment?

1. In which situations?
2. What do you talk about?
3. Has anything changed compared to before the COVID-19 pandemic?

**Open question no. 4: *(Interactions with clinicians)***

4. How do you interact with the supervising doctors of the department at the moment?

1. In which situations?
2. What do you talk about?
3. Has anything changed compared to before the COVID-19 pandemic?

**Open question no. 5: *(Interactions with patients)***

5. How do you interact with patients in the department at the moment?

1. In which situations?
2. What do you talk about?
3. Has anything changed compared to before the COVID-19 pandemic?

**Open question no. 6*: (The role of medical students)***

6a. What do you think about your role (as a medical student) in a department during the COVID-19 pandemic?

6b. Are there areas where you feel that you (as a medical student) contribute to the department? Are there areas where you feel that you (as a medical student) burden the department?

**Open question no. 7: *(Concluding remarks)***

7a. Do you have any thoughts about things that could be changed or optimized regarding your clinical clerkship at the moment?

7b. Do you have anything else you wish to state further?

**Interview protocol for doctors:**

**Open question no. 1: *(General)***

1. What is it like to have medical students participating in a clinical clerkship during the COVID-19 pandemic?

1. Has anything changed? If so, how is it different compared to (your) earlier experiences?
2. How are the medical students welcomed by their peers?
3. How are the medical students welcomed by the doctors of the department?
4. How are the medical students welcomed by the patients?

**Open question no. 2: *(Classification of clinical activities)***

2. Could you describe your last day(s) where you had medical students participating in a clinical clerkship?

1. Which activities do they participate in? What do they do on their own and what do they do in collaboration with others?
2. Are there any precautionary measures that they have to adhere to?
3. Do you experience that the recommendation to ”keep your distance” affects their activities in the clinical setting?
4. Have you experienced medical students being rejected in tasks or assignments which you believe they normally would have been able to participate in or undertake?
5. Have you experienced medical students being offered any tasks or assignments which you believe they normally would not have been able to participate in or undertake?

**Open question no. 3: *(Interactions with medical students)***

3. How do you interact with the medical students?

- 1. In which situations?
  2. What do you talk about?
  3. Has anything changed compared to before the COVID-19 pandemic?

**Open question no. 4: *(Interactions with other clinicians)***

4. How do you interact with other doctors of the department together with medical students?

- 1. In which situations?
  2. What do you talk about?
  3. Has anything changed compared to before the COVID-19 pandemic?

**Open question no. 5: *(Interactions with patients)***

5. How do you interact with patients in the department together with medical students?

- 1. In which situations?
  2. What do you talk about?
  3. Has anything changed compared to before the COVID-19 pandemic?

**Open question no. 6*: (The role of medical students)***

6a. What do you think about the role of medical students in a department during the COVID-19 pandemic?

6b. Do you feel that there are areas where medical students contribute to the department? Do you feel there are areas where medical students burden the department?

**Open question no. 7: *(Concluding remarks)***

7a. Do you have any thoughts on things that could be changed or optimized regarding medical students’ clinical clerkship at the moment?

7b. Do you have anything else you wish to state further?
